# Resurrection of an ancestral 5S rRNA

**DOI:** 10.1186/1471-2148-11-218

**Published:** 2011-07-22

**Authors:** Qing Lu, George E Fox

**Affiliations:** 1 Department of Biology and Biochemistry, 3201 Cullen Blvd., University of Houston, Houston, TX 77204-5001, USA

**Keywords:** ancestral sequence resurrection, parsimony, 5S rRNA, mutagenesis, ribosome evolution

## Abstract

**Background:**

In addition to providing phylogenetic relationships, tree making procedures such as parsimony and maximum likelihood can make specific predictions of actual historical sequences. Resurrection of such sequences can be used to understand early events in evolution. In the case of RNA, the nature of parsimony is such that when applied to multiple RNA sequences it typically predicts ancestral sequences that satisfy the base pairing constraints associated with secondary structure. The case for such sequences being actual ancestors is greatly improved, if they can be shown to be biologically functional.

**Results:**

A unique common ancestral sequence of 28 *Vibrio *5S ribosomal RNA sequences predicted by parsimony was resurrected and found to be functional in the context of the *E. coli *cellular environment. The functionality of various point variants and intermediates that were constructed as part of the resurrection were examined in detail. When separately introduced the changes at single stranded positions and individual double variants at base-paired positions were also viable. An additional double variant was examined at a different base-paired position and it was also valid.

**Conclusions:**

The results show that at least in the case of the 5S rRNAs considered here, ancestors predicted by parsimony are likely to be realistic when the prediction is not overly influenced by single outliers. It is especially noteworthy that the phenotype of the predicted ancestors could be anticipated as a cumulative consequence of the phenotypes of the individual variants that comprised them. Thus, point mutation data is potentially useful in evaluating the reasonableness of ancestral sequences predicted by parsimony or other methods. The results also suggest that in the absence of significant tertiary structure constraints double variants that preserve pairing in stem regions will typically be accepted. Overall, the results suggest that it will be feasible to resurrect additional meaningful 5S rRNA ancestors as well as ancestral sequences of many different types of RNA.

## Background

The principle of parsimony suggests that the most likely evolutionary path between two points in a sequence space is the one which requires the least number of events. Although parsimony is primarily used to construct phylogenetic trees, it and other methods such as maximum likelihood, also provides predictions of ancestral sequences. Traditionally these were ignored. This began to change with the demonstration that parsimony predictions of ancestral sequences on a viral system of known genetic history were largely correct [1]. Subsequently, there has been an increasing interest in what ancestral sequences might reveal about ancient macromolecules [2]. Thus, soon thereafter parsimony analysis was applied to reconstruct the evolutionary history of the artiodactyl ribonuclease superfamily using sequence data [3]. The resurrection of ancestral proteins and viruses [4,5] coupled with demonstration of functionality is now increasingly common and such studies are providing important insights to molecular evolution [6-8].

The primary focus of ancient sequence resurrection has been proteins [9,10] but the increasing realization that RNA played a key role in the early history of life and is intimately involved in the regulation of gene expression suggests that resurrection of ancient RNAs will likely be informative too [11]. Of particular interest is the ribosome whose history is closely intertwined with the early history of living systems [12-15]. Functional RNAs such as the ribosomal RNA, (rRNA), typically have complex secondary and tertiary structures that must be preserved over evolutionary time to facilitate function.

5S rRNA is the smallest RNA component of the large ribosomal subunit and typically contains approximately 120 nucleotides. Its secondary structure [16,17] is known and in the case of *Escherichia coli *its tertiary structure in the context of the ribosome is also available [18]. In an earlier investigation, all the sequences along the equally and most parsimonious paths between two pairs of modern 5S rRNA sequences were examined [19]. Each intermediate sequence was tested for validity as a functional 5S rRNA and it was thereby shown that some of the intermediates were not functional. Thus, some evolutionary paths through the sequence space are preferable to others despite the fact that all appear to be equally parsimonious. In order to be useful, methods for constructing ancestral sequences should to the extent possible avoid producing nodal sequences that are not biologically functional and hence are unlikely to have existed in the past. Fortunately, the usual parsimony procedure will automatically conserve sequence correlations between pairs of positions and hence the predicted nodal sequences will usually satisfy secondary structure requirements. Thus, there is good reason to anticipate that many nodal sequences will be functionally valid. Herein, we successfully test this conjecture by resurrecting an ancestral 5S rRNA sequence that is predicted by parsimony and showing that it is in fact a functional 5S rRNA.

## Methods

The experimental system employed here utilizes a plasmid encoded 5S rRNA derived from the marine bacterium *Vibrio proteolyticus *that is expressed in an *E. coli *host. This 5S rRNA was initially chosen because its 5' and 3' terminal sequence regions are identical in sequence to those of *E. coli *5S rRNA. The plasmid used, pCV251, was derived from a plasmid pKK5-1 [20], which carries a large portion of the *E. coli rrnB *operon. Additional file 1, Figure S1 illustrates the composition of plasmids pKK5-1 and pCV251. Plasmid pCV251 has two ribosomal RNA promoters, P1 and P2, a small fragment of the 16S rRNA sequence, a chemically synthesized *V. proteolyticus *5S rRNA gene and the two *rrn*B terminators, T1 and T2 [21]. Thus, the *V. proteolyticus *5S rRNA is surrounded exclusively by *E. coli *sequences thereby ensuring that all signals for correct transcription, processing, and maturation of variants of the *V. proteolyticus *5S rRNA in *E. coli *are present. The validity of the *V. proteolyticus *5S rRNA sequence and its variants is tested in the *E. coli *cellular context. There are two major assays employed for this purpose. One is an assay that allows determination of the quantitative level of each variant in three cellular fractions; e.g., total 5S rRNA, 50S ribosomal subunits, and 70S ribosomes. The other assay is a competitive growth rate assay that competes each variant with a strain carrying the wild-type *V. proteolyticus *5S rRNA. The *V. proteolyticus *5S rRNA has previously been shown to be expressed to significant levels and incorporated into ribosomes without significant detriment to growth [22]. If a variant is not functional, it will either be excluded from active ribosomes or if incorporated into large numbers of ribosomes, substantially impact cell growth rate.

The parsimony algorithm (PHYLIP version 3.5) available in GDE (Genetic Data Environment) [23] was used to construct phylogenetic trees for 32 *V. proteolyticus *5S rRNA sequences [24]. *Bacillus subtilis *5S rRNA was used as the outgroup sequence. DNAPARS, estimates phylogenies by the parsimony method using nucleic acid sequences and actually provides predicted sequences at each node on the phylogenetic tree.

*Escherichia coli *strain JM109 was used for the growth of bacteriophage M13. *E. coli *HB101 was used for rRNA preparation and in growth rate assays as lac^- ^control. *E. coli *ML401 was used in growth rate assay as a lac^+ ^competing test strain. The *E. coli *TG1 strain was supplied with Sculptor™*in vitro *mutagenesis system (Amersham Life Science Corp.) for the mutagenesis reactions. *V. proteolyticus *ATCC15338 carried the wild-type 5S rRNA. Plasmids pKK5-1 and pCV251 carry the *E. coli *5S rRNA gene and *V. proteolyticus *5S rRNA gene respectively [21].

All newly described *Vibrio *5S rRNA variants were constructed by *in vitro *mutagenesis using the manufacturer's protocol provided with the Sculptor™mutagenesis kit. The mutants were verified by dideoxynucleotide sequencing. Each *V. proteolyticus *5S rRNA variant was cloned into pKK5-1 using the HindIII sites, and subsequently transformed into *E. coli *strain JM109.

Total cellular RNA was isolated by low-pH phenol extraction [25]. Ribosomes were separated by sucrose gradient method [26]. The ribosomal subunits, 70S ribosomes, and polyribosomes were separated by ultracentrifugation at 160,000 × g at 0°C for 4 hours using a Beckman SW41 Ti swinging bucket rotor. After ultracentrifugation, all gradients were analyzed at 280 nm using an LKB detector (BROMMA) Model 2112 REDIRAC fraction collector. Fractions containing 70S ribosomes, 50S ribosomal subunits and 30S ribosomal subunits were prepared individually, followed by ethanol precipitation, and phenol/chloroform extraction.

The extent of RNA incorporation into various ribosome fractions was examined. To this end, 5S rRNAs from *V. proteolyticus *and *E. coli *were purified respectively from their total RNAs by electrophoresis on two individual 10% polyacrylamide gels [21]. The concentration of purified 5S rRNA was measured by Stratagene's EAGLE EYE™II after running on an agarose gel. Northern Blotting was performed by generating a standard 13% polyacrylamide gel, which was loaded with *V. proteolyticus *and *E. coli *5S rRNA in various calculated ratios. The RNA was then transferred from the gel to a charged nylon filter-Hybond™-N^+ ^(Amersham), which would be first hybridized with a probe (HV2) complementary to *V. proteolyticus *5S rRNA. The relative gel band density, which reflects radioactivity, was measured using a phosphoimaging system (Fuji, BAS-MP). The Macintosh program MacBas Version 2.1 was used to process the data. Subsequently, the filter was stripped completely before hybridizing a second probe (HE2) complementary to *E. coli*. The *V. proteolyticus *specific probe used (HV2, 5'-GTCCAAATCGCTATGGTTGC-3') exhibits seven nucleotide differences relative to the *E. coli *specific probe (HE2, 5'-GACCACCGCGCTACTGCCGC-3') that targets the same region of 5S rRNA., This level of difference ensures hybridization to *V. proteolyticus *5S rRNA without cross hybridization to the host *E. coli *5S rRNA [19]. Both HV2 and HE2 probes were [γ-^32^P]-ATP end-labeled. Once the radioactivity of each band from the standard gel was measured, a standard curve relating the 5S rRNA concentration ratio to probe intensity ratio of *V. proteolyticus *versus *E. coli *was constructed. The amount of each *V. proteolyticus *variant in a sample can be obtained relative to *E. coli*, e.g. as a ratio, by comparing the *V. proteolyticus *variant band density with those obtained on the standard curve. Probes HV2 and HE2 were used in this manner to quantify each variant of the *V. proteolyticus *5S rRNA in 50S subunits, 70S ribosomes, and total 5S rRNA. 5S rRNA is not present in the 30S subunits.

Competition growth assays were used to compare the growth rate of the strain carrying each variant 5S rRNA relative to a strain carrying the wild-type *V. proteolyticus *5S rRNA. *E. coli *strains HB101 and ML401 were transformed with unmodified pCV251 (control) and pCV251 carrying the variant 5S rRNA instead of the wild type *V. proteolyticus *5S rRNA (test). The growth rate difference was measured by following the ratio of cell numbers between the *Lac^- ^*HB101 (control) and *Lac^+ ^*ML401 (test) when mixed cultures were grown on indicator plates [27-31].

Growth rate assays were initiated by inoculating each strain from single colonies into 10 ml of LB medium containing 50 μg/ml ampicillin and growing at 37°C for approximately 12 hours. Subsequently, the mixture of two strains was diluted to 10^-3 ^in M9 media. Two sequential subcultures were grown. Samples were taken from each subculture, serially diluted, and plated on MacConkey indicator agar. After incubation at 37°C for 24 hours, *Lac^- ^*and *Lac^+ ^*colonies were scored on the basis of colony color (*Lac^-^*: white, *Lac*^+^: pink). The growth rate difference or selection coefficient (s) was calculated according to the following equation [26].

where N_p_(t) and N_r_(t) represent the relative number of the two competing strains at time t (hr) and s is a measure of differential growth rate (or selection coefficient) per unit time between the two strains. The strain designated by N_p _is favored, neutral, or disfavored relative to the strain designated by N_r _as s > 0, s = 0, or s < 0. A parallel experiment was performed with the strains carrying the test and control reversed.

## Results

**Figure 1:**
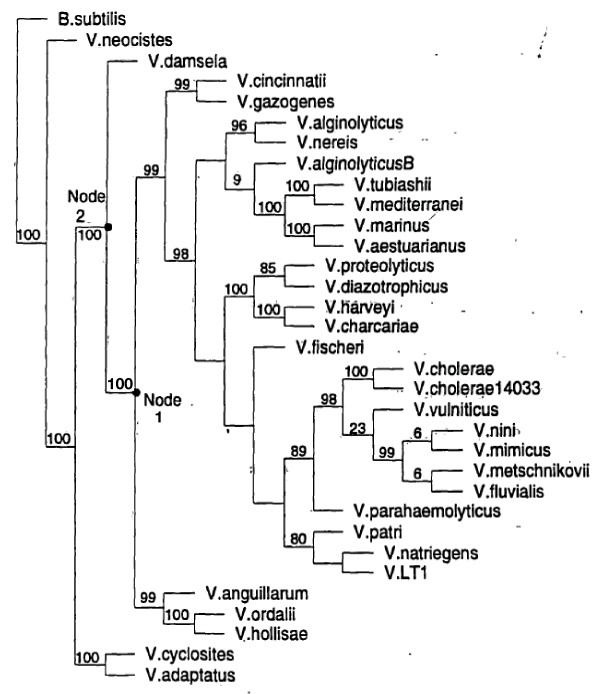
**Representative phylogenetic tree from the 100 tree set**. Nodes labeled 1 and 2 are the two ancestral nodes which were constructed and evaluated experimentally for validity as 5S rRNAs. Numbers above the branches indicate the frequency with which that branch was found in the 100 tree set.

**Figure 2:**
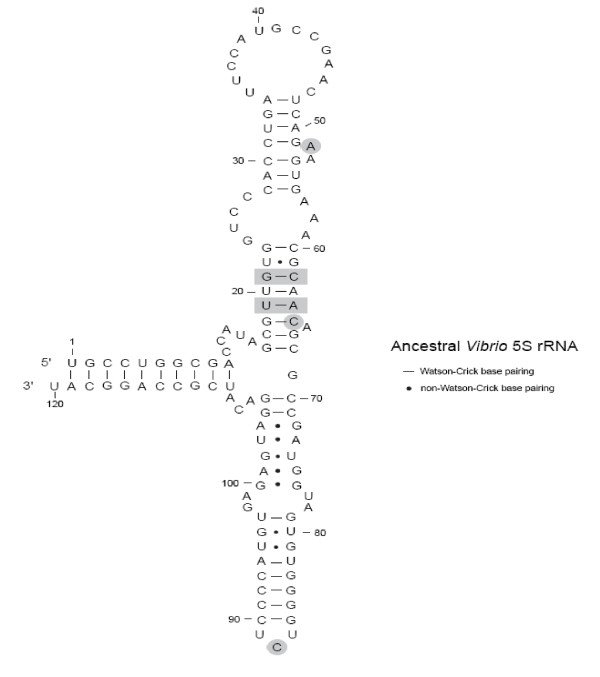
**Ancestral *Vibrio *5S rRNA Sequence**. The secondary structure is shown in an orientation that resembles that seen in the 50S subunit crystal structure. The seven highlighted bases indicate locations where the ancestral sequence (node 1) differs from the wild type *V. proteolyticus *sequence. Position numbering is the same for *E. coli *and *V. proteolyticus*.

One hundred phylogenetic trees were constructed from the *Vibrio *5S rRNA dataset using parsimony. Two ancestral nodal sequences, at node 1 and node 2, were chosen for experimental study. The sequences at both nodes were unambiguous and consistently predicted in all 100 trees. Figure 1 is a randomly chosen representative tree. There are 7 sequence differences between the modern *V. proteolyticus *5S rRNA sequence and ancestral node 1. The base changes at node 1 are A19U, U21G, U52A, A62C, U64A, U65C, and U88C as indicated on Figure 2. Of these changes, five involve positions involved in secondary structure. Thus, the 21/62 combination converts a UA pairing to a GC pairing, the 19/64 combination converts an AU pair to a UA pair and the change at position 65 converts a GU wobble pair to a Watson-Crick GC pair. In addition to the 7 changes in node 4, an eighth change, C70U, which is in a hairpin loop region, produces the node 2 ancestral sequence.

A previously constructed plasmid, [19], was used as the starting point for the mutagenesis because it already had three of the required base changes, A19U, U52A, and U64A. This plasmid produces an RNA that is functional in the *E. coli *cellular context. A series of consecutive mutagenesis reactions were performed to construct the final two plasmids carrying the complete node 1 and node 2 sequences. This mutagenesis process resulted in the formation of two intermediates sequences. Relative to the *V. proteolyticus *wild type 5S rRNA, the first intermediate had changes A19U, U21G, U52A, and U64A, while the second had changes A19U, U21G, U52A, A62C, U64A, and U65C. In addition to the two ancestral nodal sequences and the two intermediate sequences, two single point mutations (G33C and C49G) and one double mutant (G33C; C49G) were made in the work described here. Positions 33 and 49 are pairing partners in a duplex region of 5S rRNA and are not associated with the resurrection. After mutagenesis, each variant was confirmed by DNA sequencing and cloned into the expression vector as described in the methods. Table 1 shows the results of quantization and growth rate analysis for these seven mutants. In addition, eleven other relevant variants have been previously studied [19,32] and are included in Table 1.

**Table 1:** Properties of various *V. proteolyticus *5S rRNA variants discussed in the text

	Variant	Reference	Rate Diff	% RNA	% 70S	%50S	Phenotype
1	A19U	17	0.02	<1	<1	<1	Blue
2	G33C	This paper	0.04	>50	<1	<1	Red
3	U35A	30	0.02	>50	15	16	Green
4	C49G	This paper	-0.01	>50	<1	<1	Red
5	U52A	17	0.00	>30	>30	>30	Green
6	U64A	17	0.05	>20	<1	<1	Red
7	U65C	30	0.03	>50	>50	>50	Green
8	C70U	30	0.05	9	8	6	Blue/Green
9	U88C	30	0.03	>50	23	22	Green
10	A19U;U52A	17	0.02	>30	>30	>30	Green
11	A19U;U64A	30	0.02	>50	48	43	Green
12	U21G;A62C	30	-0.01	33	23	25	Green
13	G33C;C49G	This paper	-0.01	>50	>50	>50	Green
14	A19U;U52A;U64A	17	0.03	>30	>30	>30	Green
15	A19U;U21G;U52A;U64A	This paper	0.02	15	5	8	Blue/Green
16	#15 plus A62C &U65C	This paper	0.04	>50	>50	>50	Green
17	#16 + U88C	This paper	0.04	23	26	25	Green
18	#17 + C70U	This paper	0.04	2	1	<1	Blue
19	*V. proteolyticus*	This paper	0.00	38	38	34	Green

None of the constructs had a substantial affect on the growth of the host strain. However, by examining the accumulation levels of each construct in the total RNA pool and the various ribosome fractions each construct can be classified into one of three major categories referred to as green, red and blue. The green variants are considered to be functional. They are extensively accumulated and incorporated into 50S ribosomal subunits and 70S ribosomes without significant detrimental effect on cell growth rate. The red variants are extensively accumulated in the total 5S rRNA pool but not significantly incorporated into 50S ribosomal subunits or 70S ribosomes. These variants are considered to be non-functional. The blue variants such as A19U accumulate poorly in the total 5S rRNA pool, apparently because the RNA has become unstable. Typically, these variants are expected to be non-functional but in some cases they may actually be functional as they are observed to accumulate at low but nevertheless similar levels in 50S ribosomal subunits and 70S ribosomes. The single base change variant C70U is an example of a blue variant of this type.

The node 1 sequence, which is the predicted common ancestor of 28 *Vibrio *5S rRNAs, was in the green category and can therefore be considered to be a valid 5S rRNA. Likewise all the intermediates that did not disrupt secondary structure were also found to be functional in the *E. coli *context. The additional base change required to create the node 2 sequence resulted in loss of stability such that it was not possible to unambiguously determine whether this nodal sequence would be functional or non-functional, if it accumulated at higher levels. However, it is best concluded that this nodal sequence is likely not a valid ancestor.

## Discussion

It is inherent in the concept of an ancestral sequence that a true ancestor would have been functional in the cellular context that existed at the time. Thus, if a predicted ancestor when resurrected is functional in the modern cellular context, it is far more likely to be a realistic ancestor than one that is not. One can argue that viability of a true ancestor might be lost following resurrection due to changes in the cellular context. In the present instance, we believe this is unlikely to be the case. In prior work, [22] the wildtype sequences of ten different species of modern *Vibrio *5S rRNAs were tested for functionality in the *E. coli *cellular environment. All, were accumulated in ribosomes without significant detriment to growth rate (most were actually slightly favorable) and therefore regarded as functional in the *E. coli *cellular environment. The evolutionary distance between *E. coli *and any of these 10 *Vibrio *species clearly exceeds the distance between the modern *Vibrio *5S rRNAs and the ancestors reconstructed here.

The node 1 5S rRNA was found to be functional in the *E. coli *cellular context. This RNA has seven base changes relative to the modern *V. proteolyticus *5S rRNA Given the low probability that this number of changes could be randomly introduced without disrupting function [33], this should be regarded as a successful resurrection of a realistic ancestor. Of these changes, all have previously been studied as individual point mutations, or in the case of those involving base pairing, as appropriate double mutants, Table 1. When made individually, U52A, U65C and U88C, produced functionally valid 5S rRNAs. Likewise, when both partners in the 19/64 and 21/62 base pairs are changed simultaneously, the resulting 5S rRNA was a functionally valid 5S rRNA. Thus, the individual changes that comprise the ancestral sequence all produce valid 5S rRNAs too. This reaffirms the earlier conclusion [32] that variants which occur frequently in a local region of the 5S rRNA structure space (these seven do) are largely independent of one another and hence can occur separately or together in many sequences, e.g. in this case in the predicted ancestral sequence without disrupting function.

When only one residue in a base pair was changed, e.g. A19U or U64A the individual point mutations did not produce a functional 5S rRNA whereas changing both positions in unison did. Since single changes are more likely to occur than double changes one might expect paired regions to change more slowly than other regions. However, this need not be the case. It was found in the earlier pathway studies [16] that one could bypass the double mutant by making other variants first. In addition, the success of the three double mutants examined here in producing valid 5S rRNAs indicates that there is a very high probability that a double mutant that preserves secondary structure will be accepted, if the bases that comprise the pair are not involved in tertiary interactions. In contrast, a single mutation in a non-paired region may be significantly less likely to be accepted. Thus, the overall rate of acceptance may not be dissimilar even though double mutants are less common. Also, and perhaps more importantly, it should be appreciated that one can potentially transition from one standard pair to another via a GU wobble or GA intermediary.

Although the node 1 sequence is regarded as a valid ancestral sequence, a single additional base change, C70U, made the RNA sequence represented by node 2 unstable. Since the node 2 RNA does not accumulate to a significant extent and is absent from ribosomes, it is at best very suspect as a valid ancestor. As a separate variant, the C70U change alone, Table 1, is also unstable in the *E. coli *cellular context. It does, however, accumulate in ribosomes enough to suggest that it is likely functional, if it can avoid premature degradation. Thus, the behavior of the multiple variant at node 2 is again strongly influenced by the behavior of the individual variants that comprise it.

From the perspective of the present study, the appropriate conclusion is that node 2 is most likely not a valid ancestor. When one traverses from node 1 to node 2, the new nodal sequence is based on the addition a single additional 5S rRNA sequence, *Vibrio damsela *whose 5S rRNA has C70U. However, relative to node 2, this 5S rRNA has three additional base changes, A25U, U35A, and C88U. Unless it was erroneously determined, the *V. damsela *sequence is a valid 5S rRNA. Thus, the comparison suggests that U25, A35, or U88 in some combination or separately compensate for the negative effect of C70U in order to make this 5S rRNA functional. The availability of additional sequences related to *V. damsela *might have improved the prediction at node 2. The results presented here point out the danger of allowing predictions to be strongly influenced by a single sequence.

## Conclusions

In summary, it has been demonstrated that an ancestral 5S rRNA sequence predicted by parsimony can serve as a functional RNA and thus can be regarded as reasonable approximations of a true ancestral sequence. However, a second predicted ancestral sequence did not pass the test of convincing functionality. In both cases, the functional behavior of the ancestral sequence could be largely anticipated based on the behavior of individual base or base pair changes that comprised the predicted ancestral sequence. A strength of the parsimony approach in the present context is that by its nature, meaningful secondary structure will be preserved because paired positions will typically exhibit co-variation when numbers of sequences are aligned. In the future, it should be possible to resurrect other 5S rRNA ancestors as well as other interesting RNAs such interesting regions of the large ribosomal RNAs.

## List of Abbreviations

XN, e.g. U88: Uracil at position 88; XNY, e.g. U21G: Uracil at position 21 changed to guanosine; 5S rRNA: 5S ribosomal RNA.

## Authors' contributions

GEF conceived of the study, participated in the interpretation of the results, and prepared the final manuscript. QL designed the experimental system and did all the experimental work, and participated in the interpretation of the results and prepared the first draft of the manuscript. All authors read and approved the final manuscript

## Supplementary Material

Additional file 1 **Composition of plasmids pKK5-1 and pCV251**. Diagrams showing details of plasmids pKK5 and pCV251. Click here for file
