# Supplementary material for: Resurrection of an ancestral 5S rRNA
Source: BMC Evol Biol. 2011 Jul 22;11:218. doi: 10.1186/1471-2148-11-218 (PMC3161009; doi:10.1186/1471-2148-11-218)
Supplement: Additional file 1 — Composition of plasmids pKK5-1 and pCV251. Diagrams showing details of plasmids pKK5 and pCV251. [file 1471-2148-11-218-S1.DOCX]

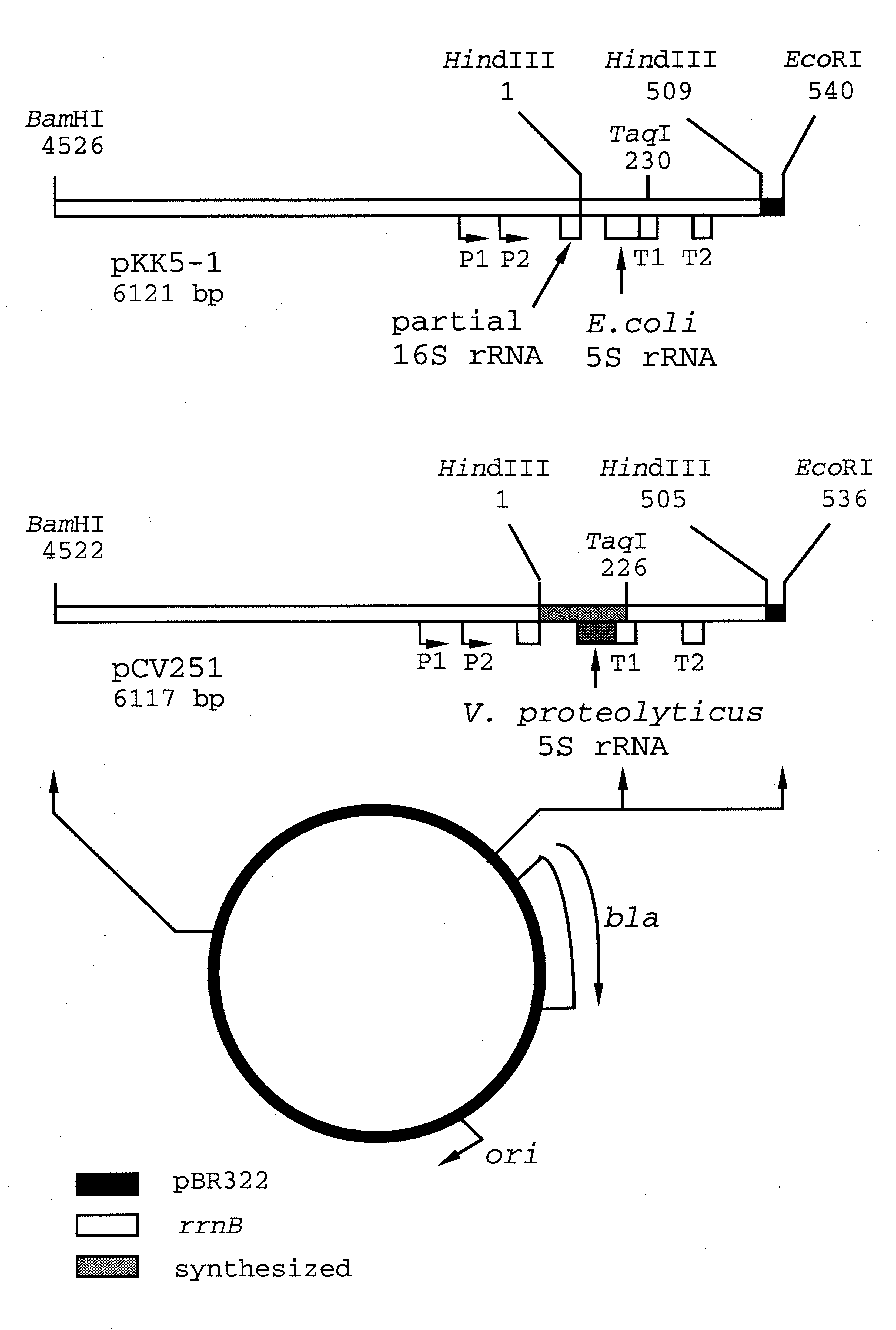


**Additional File 1: Figure S1**

**Comparison of Plasmids pKK5-1 and pCV251.** Both plasmids contain the *E. coli rrn*B operon promoters P1 and P2, an 85 bp fragment from the 5' end of 16S rRNA, the complete *V. proteolyticus* 5S rRNA gene, terminators T1 and T2, and flanking sequences. In pCV251 the *E. coli* 5S rRNA gene of pKK5-1 is replaced with a chemically synthesized *V. proteolyticus* 5S rRNA [21]. The first A in the *Hind*III endonuclease site (AAGCTT) upstream of the 5S rRNA gene is denoted as nucleotide number 1. Abbreviations: *bla*, β-lactamase (ampicillin resistance); *ori*, origin of replication; P1 and P2, promoters 1 and 2; T1 and T2, terminators 1 and 2.
